# Don’t forget to mind the mind: a prospective cohort study over 12 months on mental health symptoms in active professional male footballers

**DOI:** 10.1186/s13102-024-01005-1

**Published:** 2024-10-14

**Authors:** Lervasen Pillay, Dina C. Janse van Rensburg, Gopika Ramkilawon, Thor Einar Andersen, Gino Kerkhoffs, Vincent Gouttebarge

**Affiliations:** 1 Amsterdam UMC Location University of Amsterdam, Department of Orthopaedic Surgery and Sports Medicine, University of Amsterdam, Meibergdreef 9, Amsterdam, AZ 1105 The Netherlands; 2 Section Sports Medicine, Faculty of Health Sciences, University of Pretoria, Pretoria, South Africa; 3 Department of Statistics, University of Pretoria, Pretoria, South Africa; 4 Oslo Sports Trauma Research Center, Norwegian School of Sport Sciences, Oslo, Norway; 5 Academic Center for Evidence-Based Sports Medicine (ACES), Amsterdam, The Netherlands; 6 Amsterdam Collaboration on Health & Safety in Sports (ACHSS), IOC Research Center of Excellence, Amsterdam, The Netherlands; 7 Amsterdam Movement Sciences, Aging & Vitality, Musculoskeletal Health, Sports, Amsterdam, The Netherlands; 8 Football Players Worldwide (FIFPRO), Hoofddorp, The Netherlands

**Keywords:** Mental health, Male football, Football distress, Football anxiety, Football depression

## Abstract

**Aims:**

We examined the prevalence and incidence of mental health symptoms (MHS) in active professional male footballers over a 12-month period and investigated if MHS was associated with severe injuries or surgeries.

**Participants and methods:**

Football Players Worldwide (FIFPRO) affiliated national unions invited active professional male football players to participate in the study. MHS was operationalised in symptoms of anxiety, disordered eating, depression, distress, sleep disturbance, alcohol misuse and drug misuse, all being assessed with validated questionnaires.

**Results:**

Of the 101 participants enrolled, the prevalence of distress was 53% and MHS was between 6% for drug misuse and 48% for alcohol misuse. The incidence of distress was 29% and MHS ranged from 1% for anxiety to 11% for sleep disturbance. At baseline, players suffering from injury/surgery were more likely to report depression (OR 1.35; 95%CI 1.10–1.70) and disordered eating (OR 1.22; 95%CI 1.02–1.47). At follow-up, players who suffered injury or surgery were inclined to report distress (OR 2.15; 95%CI 1.26–4.31) and drug misuse (OR 2.05; 95%CI 1.01–4.04).

**Conclusion:**

There seems to be a greater prevalence of MHS in active professional male footballers than in the global population and other sports. After severe injury/surgery, the risk of developing MHS is increased, confirming that healthcare professionals should be aware of the mental health of injured players.

**Supplementary Information:**

The online version contains supplementary material available at 10.1186/s13102-024-01005-1.

## Introduction

The World Health Organisation’s (WHO) World Mental Health Report from 2022 mentions that one in eight people are living with a mental health disorder with anxiety and depressive disorders as the most prevalent conditions [1]. A mental health disorder is a condition that fulfils diagnostic criteria according to the Diagnostic and Statistical Manual of Mental Disorders 5 (DSM-V) [2]. The International Olympic Committee (IOC) defines mental health symptoms (MHS) as “any adverse thought, feeling, behaviour and/or psychosomatic symptom that might lead to subjective distress or functional impairments in daily life, work and/or sport” [3]. Literature has shown that physical activity improves MHS, especially anxiety and depression [4]. Contrastingly, a systematic review has suggested that football participation among active and retired footballers and referees does not appear to improve MHS [5]. Although elite sportspersons are usually physically active, they are not immune from being affected by MHS or disorders [6]. Recently, research relating to the mental health of elite-level athletes has increased. Commonly, anxiety, distress, sleep disturbance, depression and alcohol misuse are MHS encountered in elite athletes [7]. Stressors for MHS may be sport-related or off-the-field psychosocial issues and include, but are not limited to (1) injuries and surgery [8], (2) performance pressure [9], (3) transition from semi-elite to elite and from elite to retirement [9, 10], (5) travel [11], (6) match fatigue and poor recovery [12, 13] and (7) personal challenges (including adverse life events, racism and discrimination) [9]. A previous prospective cohort study of football players from five European countries over 12 months explored if there was an association between various stressors (negative life events and conflicts with coaching staff) and MHS [14]. The authors found that MHS ranged between 12% (distress) and 37% (anxiety and depression). However, active professional male footballers need more scientific studies on MHS and specific stressors like severe injuries and/or surgeries. These injuries and/or surgeries may onset a plethora of MHS due to (1) prolonged return to play, (2) complications during rehabilitation, (3) recurrent injuries and, (4) complications during and after surgery. Symptoms of, e.g., distress, anxiety and depression may present as the player begins feeling pressured to perform on the field and is financially and contractually affected by the injury.

Therefore, we first aimed to investigate the prevalence and incidence of MHS over 12 months in active professional male footballers. Secondly, we aimed to investigate whether there was any association between MHS and specific stressors (severe injuries and/or surgeries).

## Materials and methods

### Design

A prospective cohort study of an observational nature was conducted and included a review after 12 months. The Strengthening the Reporting of Observational Studies in Epidemiology statement was used to guarantee the quality of reporting [15]. Ethical approval for the study was provided by The Medical Ethics Review Committee of the Amsterdam University Medical Centers (Amsterdam UMC, location AMC) (Drake Football Study: NL69852.018.19 | W19_171#B202169). The study was conducted in accordance with the Declaration of Helsinki (2013). The age group of the cohort (as described in the Participants section below) was chosen as the findings of this study will form part of future planned studies in a 10-year follow-up of active male footballers transitioning into retirement.

### Participants

The population under investigation were active professional male footballers. Football Players Worldwide (FIFPRO) utilised their national unions which were affiliated with them, to recruit participants. Criteria for participants to be included were: (a) of male gender; (b) a professional footballer; (c) aged between 24 and 30 years, and (d) literate in English or French. We defined a professional male footballer as one who (i) improves performances by regular training, (ii) competes in the first or second tier of the national league, and (iii) Trains and competes in football as a way of life, dedicating most of their time in all or several days with little time spent doing other activities (i.e. leisure or professional) [16]. We calculated that to achieve a power of 80% at a confidence interval (CI) of 95% and absolute precision of 5% assuming a population proportion (prevalence of MHS) of 10%, we required at least 100 participants at baseline [17].

### Mental health symptoms (dependent variables)

We used aspects from the athlete’s forms 1 and 2 of the IOC Sport Mental Health Assessment Tool 1 (SMHAT-1), specifically, symptoms of anxiety, disordered eating, depression, distress, sleep disturbance, alcohol misuse and drug misuse, to operationalise mental health symptoms [18]. The baseline questionnaires asked about MHS experiences in the preceding four weeks.

Distress was evaluated using the Athlete Psychological Strain Questionnaire (APSQ). Scoring was done using a scale consisting of 5 points (ranging from “none of the time” to “all of the time”) [19]. It has been validated in athletes (internal consistency [IC]: 0.5–0.9; criterion-related validity [CV]: area under the receiver operating characteristics [ROC] curve > 0.9) [19, 20]. Adding the scores of the 10 items of this tool, a score ranging from 10 to 50 was obtained. A score of 17 or more indicates an increased risk for distress and suggests screening for other MHS [19, 20].

The 7-item General Anxiety Disorder-7 (GAD-7) was used to evaluate symptoms of anxiety. Scoring was done using a scale of 4 points (ranging from “not at all” to “nearly every day”) [21]. Validation of this tool has been done in different populations and multiple languages in Europe (IC: 0.9; test-retest reliability: 0.8; CV: sensitivity 0.9, specificity 0.8, area under the ROC curve > 0.9) [21]. Adding the scores of the seven items of this tool, a score ranging from 0 to 21 was obtained. A total score of 10 or more indicated moderate anxiety [21].

The Patient Health Questionnaire-9 (PHQ-9) was used to evaluate the presence of symptoms of depression. Scoring was done using a scale of 4 points (ranging from “not at all” to “nearly every day”) [22, 23]. Validation of this tool has been done in different populations and multiple languages in Europe (IC: >0.8; CV: sensitivity > 0.8, specificity > 0.8, area under the ROC curve > 0.9) [22, 23]. Adding the scores of the nine items of this tool, a score between 0 and 27 was obtained. A score of 10 or more indicated moderate depression [22, 23].

To evaluate the presence of disordered eating, the Brief Eating Disorder in Athletes Questionnaire (BEDA-Q) was used [24]. The BEDA-Q has been validated in athletes (IC: >0.8; CV: sensitivity > 0.8, specificity > 0.8, area under ROC Curve > 0.7) [24]. Adding the scores of the first six items of this tool, a score between 0 and 18 was obtained. A score of 2 or more indicated disordered eating [24].

Sleep disturbance was evaluated with the shortened Athlete Sleep Screening Questionnaire (ASSQ) with scoring being done using scales consisting of 4- and 5 points [25, 26]. The ASSQ has been validated in athletes (IC: >0.7; test-retest reliability: >0.8; CV: sensitivity > 0.8, specificity > 0.9) [24, 25]. Adding the items with a score of 8 or more, a score between 1 and 17 was obtained. This indicated moderate sleep disturbance [25, 26].

The level of alcohol consumption was evaluated using the validated 3-item Alcohol Use Disorders Identification Test (AUDIT-C) [27]. Validation of this tool has been done in different populations and multiple languages in Europe (test-retest coefficients: 0.6–0.9; CV: area under ROC Curve 0.70 < 1.0) [27, 28]. Adding the scores, a score between 0 and 12 was obtained. A score of 3 or more indicated alcohol misuse [27].

Drug(s) misuse in the preceding three months was evaluated using the Cutting down, Annoyance by criticism, Guilty feelings, and Eye-openers Adapted to Include Drugs (CAGE-AID) tool. Scoring was done dichotomously using “yes” or “no” [29, 30]. Validation of this tool has been done in different populations and multiple languages in Europe (reliability: >0.9; sensitivity: >79%; specificity: >97%) [29, 30]. Adding item scores, a score between 0 and 4 was obtained. A score of 2 or more indicated drug misuse [29, 30].

### Specific stressors - severe injuries and/or surgeries (independent variables)

Athletes were asked to identify whether they had experienced severe injuries and/or surgeries of the lower extremities and to identify the specific joint and side affected. Our study defined a severe injury as an injury involving a specific joint (hip, knee or ankle) sustained at training or a match leading to the absence from training or matches for longer than 28 days [31]. Participants were requested to obtain this information from their medical file or team medical staff. There was no time frame related to injuries of surgeries and they could have been sustained anytime in their careers.

### Procedures

A baseline and follow-up questionnaire (Appendix A), including all study variables, were developed in English and French utilising an electronic system (CastorEDC, CIWIT B.V, Amsterdam, the Netherlands). Descriptive variables were collected in the baseline questionnaire. These included: height, age weight,, study and occupational activity, level of education, field position, seasons played, level of football, history of hospitalisation, history of smoking and medication use (pain medication, sedatives, antidepressants, sleep aids). A history of mental health disorders and mental health status was evaluated using the validated [32] Patient-Reported Outcomes Measurement Information System Global Health short form mental health subscale (PROMIS-GMH) [32].

An information leaflet relating to the study was sent via email by FIFPRO and affiliated national unions to players to consider participating in the study. For privacy reasons, these procedures were hidden from the principal researcher. Interested participants provided informed consent electronically. Once this was obtained, they were allowed to access the baseline questionnaire (which took 15–20 min to complete). Follow-up questionnaires were sent 12 months later – these were the same as the baseline questionnaires (excluding demographic information and questions relating to injuries and surgeries and MHS relating to the previous 12 months after baseline). Responses were allocated codes and any identifying information was redacted to anonymise participants. This was to ensure confidentiality and privacy. Responses were saved after completion on a secured server. Only the principal investigator had access to this information. All participation was voluntary and no remuneration or reward was given to entice participation. Baseline data was collected early in the season.

### Statistical analyses

The statistical software “R software” (version 4.1.3; http://www.r-project.org/) was used for all data analyses. We performed descriptive analyses for all the required variables (standard deviation (SD), mean, range and frequency).

For our first aim, we calculated prevalence with the adjusted Wald method at a confidence interval (CI) of 95% [17]. From the baseline data, prevalence was presented as the number of participants with mental health symptoms [17]. Incidence was presented as the number of participants with newly determined mental health symptoms after 12 months relative to participants free from mental health symptoms at baseline [16].

For our second aim, logistic regression analysis was performed at baseline for all participants and at 12-month follow-up (for participants free from mental health symptoms and injuries and/or surgeries at baseline). Mental health symptoms represented the dichotomous dependent variable, and injuries and surgeries represented the continuous independent variables. These results were used to calculate the odds of developing mental health symptoms with a history of injuries or surgeries and were presented as odds ratios (OR) with a 95% CI.

## Results

### Participant demographics and characteristics

A total of *n* = 101 participants (Table 1) completed the baseline questionnaires with a 79.2% (*n* = 80) response rate to the review questionnaire 12 months later. Mean age was 26.5 years and 56.4% played at the highest national level for 7.6 seasons. The PROMIS-GMH T-score was 53.2 (indicating that active professional male footballers’ mental health was similar to the average United States reference population). Smoking was reported by 6.9% (*n* = 7) of participants in the previous 30 days (ranging from 1 to 20 cigarettes). Prescription pain medication was used by 36.7% (*n* = 37) either once every 3 weeks or once a year at a similar frequency of use for the 52.5% (*n* = 53) who used over-the-counter pain medication. Prescription sleeping tablets medication was used by 4.0% (*n* = 4) once a year or less while 9.0% (*n* = 9) used over-the-counter sleeping aids either daily or yearly. No participants reported using antidepressant medication. A total of 5.0% (*n* = 5) of players admitted to a diagnosis of a mental health condition (anxiety and depression) and 8.0% (*n* = 8) had first and second-degree family members who had a history of a mental health condition being diagnosed (anxiety, depression and schizophrenia).

### Prevalence and incidence of mental health symptoms

We found the prevalence of MHS at baseline amongst active professional male footballers to be 52.5% with distress, followed by 47.5% alcohol misuse, 16.8% sleep disturbance, 12.9% disordered eating, 7.9% anxiety, 7.9% depression, and 5.9% drug misuse. The incidence of MHS at the 12-month follow-up was 29.1% with distress, followed by 10.7% with sleep disturbance, 6.8% with disordered eating, 5.7% with alcohol misuse, 2.1% with drug misuse and 1.1% with anxiety. There was no incidence reporting for depression symptoms (Table 2).

### Association between mental health symptoms and injuries and surgeries at follow-up

At baseline (Table 3), we found a significantly increased odds ratio of those who sustained one or more severe injuries compared to those who did not, developing the following MHS: 1.35 (95% CI; 1.10–1.70) for depression and 1.22 (95% CI; 1.02–1.47) for disordered eating. At 12-month follow-up (Table 4), we found the following significantly increased odds ratio of those who sustained one or more severe injuries compared to those who did not, in developing the following MHS: 2.15 (95% CI; 1.26–4.31) for distress and 2.05 (95% CI; 1.01–4.04) for drug misuse.

**Table 1:** Demographics and characteristics of participants (*n* = 101)

		*n*	%	SD
Demographics	***Age***	26.5		1.7
	***BMI***	23.8		4.2
Football characteristics	***Seasons played***	7.6		2.6
	***Playing position***			
	Goalkeeper	23	22.8	
	Defender	42	41.6	
	Midfielder	25	24.8	
	Forward	11	10.9	
	***Career Level***			
	Highest national level	57	56.4	
	Second highest national level	32	31.7	
	Other levels	12	11.9	
PROMIS-GMH T-score		53.2		10.0
Smoking	***Last 30 days***	7	6.9	
Medication	***Prescription analgesia***	37	36.6	
	***Over-the-counter analgesia***	53	52.5	
	***Prescription sedatives***	4	3.9	
	***Over-the-counter sleeping aids***	9	8.9	
	***Prescription antidepressants***	0	0.0	
Diagnosed Mental Health Conditions	***Participant***	5	4.9^(Anx; Dep)^	
	***Family members***	8	8.0^(Anx; Dep; Sch)^	

n = number; SD = Standard Deviation; %=percentage; BMI = Body Mass Index; PROMIS-GMH = Patient Reported Outcomes Measurement Information System Global Mental Health; Anx = anxiety; Dep = depression; Sch = schizophrenia

**Table 2:** Prevalence and incidence of mental health symptoms (*n* = 101)

	Baseline prevalence		12-month incidence	
	*n*	%	*n*	%
Distress	53	52.5	14	29.2
Anxiety	8	7.9	1	1.1
Depression	8	7.9	0	0.0
Sleep Disturbance	17	16.8	9	10.7
Disordered Eating	13	12.9	6	6.8
Alcohol Misuse	48	47.5	3	5.7
Drug Misuse	6	5.9	2	2.1

n = number; %=percentage

**Table 3:** Association between mental health symptoms and severe injuries and/or surgeries at baseline

Mental health symptom	Injuries			Surgeries		
	*n*	OR	95% CI	*n*	OR	95% CI
Distress	158	1.07	0.92–1.24	37	1.07	0.73–1.58
Anxiety	22	0.99	0.73–1.26	5	0.96	0.39–1.77
Depression	***47***	1.35*	***1.10–1.70***	11	1.64	0.94–2.76
Sleep Disturbance	49	1.02	0.83–1.21	9	0.85	0.44–1.40
Disordered Eating	***58***	1.22*	1.02–1.47	8	0.95	0.48–1.59
Alcohol Misuse	116	0.92	0.78–1.06	23	0.71	0.45–1.05
Drug Misuse	24	1.14	0.87–1.44	2	0.62	0.11–1.52

n = number; CI = Confidence Interval; OR = odds ratio. *=significant association. Note: Baseline total injuries = 278; Baseline total surgeries = 67

**Table 4:** Association between mental health symptoms and severe injuries and/or surgeries at 12-month follow-up

Mental health symptom	Injuries			Surgeries		
	*n*	OR	95% CI	*n*	OR	95% CI
Distress	***36***	2.15*	***1.26–4.31***	12	39.39	0.31-5037.16
Anxiety	0		NA	0		NA
Depression	0		NA	0		NA
Sleep Disturbance	11	1.21	0.72–1.89	3	1.33	0.39–3.41
Disordered Eating	10	1.32	0.77–2.11	2	1.19	0.24–3.25
Alcohol Misuse	18	1.39	0.91–2.17	6	2	0.79–5.51
Drug Misuse	***6***	2.05*	***1.01–4.04***	0		NA

n = number; CI = Confidence Interval; OR = odds ratio; *=significant association. Note: Follow-up total injuries = 47; Follow-up total surgeries = 12

## Discussion

Our findings revealed that the prevalence of distress was 53% and MHS in active professional male footballers ranged from 6.0% for drug misuse to 48.0% for alcohol misuse, while the 12-month incidence of distress was 29.0% and MHS ranged between 1.0% for anxiety to 11.0% for sleep disturbance. Our baseline questionnaire results found that active professional male footballers who reported sustaining a severe injury and/or required surgery previously were 1.6 times more likely to report MHS. However, when we administered the same questionnaire 12 months later, we found the results not to be statistically significant. Still, our findings suggest that a severe injury and/or surgery history is likely a risk factor for MHS among active professional male footballers.

### The perspective of these findings

This study found that the prevalence of distress was 53.0% and MHS ranged from 6.0% for drug misuse to 48.0% for alcohol misuse, while the incidence of distress was 29.0% and MHS ranged from 1.0% for anxiety to 11.0% for sleep disturbance. Since we operationalised the components of the SMHAT-1 tool, we reported results relating to distress using the APSQ, which is athlete-specific. The APSQ also serves as a triage tool to further screen for MHS among active professional male footballers. The World Health Organisation’s World Mental Health Report in 2022 [33] reports that the global prevalence in males 20 years and older of disordered eating, anxiety and depression ranges between 0.2% and 4.0%. Regional population prevalence ranges between 1.0 and 8.0% in Africa/Middle East, 0.2% to more than 10.0% in Europe, 0.1–8.0% in Asia Pacific and 0.1–10.0% in the Americas. Europe regions range between 0.1% and 10.0% in Northern, Western and Southern Europe and 0.1% and 6.0% in Eastern Europe [34]. We assume there is a higher prevalence of MHS amongst active professional male footballers compared to the global and regional prevalence, but this difference might be due to the use of variable screening tools.

A previously reported cross-sectional analysis study of active professional male footballers from European professional leagues [8], where two measures over 6 months were obtained, reported MHS prevalence ranging between 10.0% and 58.0%. Findings of an observational cross-sectional study amongst both genders of active professional football players in Denmark reported a prevalence of MHS ranging between 3.0% and 18.0% [35]. A study amongst active professional Japanese male footballers reported a prevalence of MHS ranging between 3.1% and 9.4% [36]. In comparison, an Australian cohort study reported a prevalence ranging between 5.0% and 52.0% amongst the active professional male footballers subset [37]. We assume that the wide variance of the prevalence of MHS in active professional male football players may be due to methodological differences: (a) mixed gender cohorts (gender MHS prevalence difference) [37], (b) different validated tools used (e.g., SMHAT-1 vs. 12-item General Health Questionnaire), (c) different stressors investigated (e.g., injuries and/or surgeries vs. other life events) [8, 37] and (d) different country cohorts (e.g., global regional MHS prevalence difference) [34]. A previous prospective observational cohort over 12 months with three repeated measures on retired professional male footballers, reported an increase in MHS incidence after a negative life event (e.g., spousal death, divorce) ranging between 11.0% and 29.0% [38]. We postulated that the difference in the incidence of different MHS in the retired compared to the active male football player may be attributed to several factors [39]: (a) lack of competitive external stimulus, (b) change in life priorities, (c) more spare time to engage in activities which may result in substance misuse and (d) reduction in activity intensity and frequency losing the depressive-protective effect of exercise. Our findings are similar to other studies revealing a higher prevalence [8, 37] and incidence [14] of MHS among active professional male footballers compared to the global and regional populations.

An Australian study of high-level athletes in multiple sports found that in the male cohort, the prevalence of depression was 10.3%, disordered eating 0.4%, distress 14.6% and anxiety 3.3% [40]. A study amongst Swedish male athletes who participated in multiple sports found the prevalence of depression and anxiety at 5.6% and alcohol misuse at 27.7% [41]. The variability in prevalence findings may be due to the various validated tools used in these studies compared to the tools utilised in our study.

As in a previous study [8], we also found increased odds of developing MHS after sustaining injuries and/or surgeries, confirming that those can be seen as a major stressor in elite sports. However, odds ratios were different for the subsets of MHS – suggesting that there may also be an association between the type of injury and/or surgeries and match-time loss (e.g. Anterior Cruciate Ligament Rupture compared to ankle sprain) sustained (we did not investigate this association further). An elite active professional Swedish male football player study found that MHS was associated with performance pressure, social media and injuries [42] suggesting multifactorial contributing factors According to our knowledge, our findings of a significant association and increased odds of developing disordered eating and drug misuse after severe injuries and/or surgeries have not been previously reported in the literature.

Our study found that 5.0% of players have been previously diagnosed with anxiety and/or depression and not medicated, 18.0% smoked cigarettes (a 3.0% prevalence has been previously reported [8]), 4.0% used prescription sedatives and 9.0% used sleeping aids. The increased prevalence of MHS we found may explain the use of mood-altering substances [43, 44].

### Clinical and research implications of the findings

Since MHS have multifactorial contributing factors, clinicians should always have a high suspicion of MHS in players, especially after sustaining severe injuries and/or surgeries. Using validated screening tools is vital to gain an unbiased clinical suspicion of diagnosis and have a management plan for the condition [45]. The IOC Sport Mental Health Recognition Tool (SMHRT-1) [18] can be utilised as an awareness and screening tool after injuries and/or surgeries. Furthermore, it can assist in identifying MHS early so further assessment and intervention can proceed to enable optimal function in all spheres of life, including performance as a professional football player. The IOC SMHAT-1 [18] tool can be utilised to identify those requiring further mental health clinical assessment. Furthermore, using common validated tools will support future research so systematic reviews and meta-analysis results can provide a more accurate estimation of MHS in active professional male football players and destigmatising MHS. While most existing literature had reported on MHS prevalence only, we also reported on incidence. Incidence reporting can help identify which MHS can develop after specific stressors. Clinicians may therefore be more inclined to screen for MHS expeditiously and have a mental health management plan. Our findings of previously unreported high odds of developing disordered eating and drug misuse after severe injuries and/or surgeries require further investigation. It may explain the prevalence of drug misuse and prohibited substance use prevalence and incidence in active professional male football players.

### Strengths and study limitations

This study is the first study to report on findings related to mental health symptoms and their association with severe injuries and/or surgeries in active professional male football players in Europe. A strength was that our reporting on prevalence, incidence and associations of MHS with severe injuries and/or surgeries revealed new information, i.e. the incidence and odds of developing disordered eating and drug misuse. The 12-month follow-up questionnaire received an 80% response rate which is greater than the mean of 50%, typical for questionnaire-based studies [46]. The aims of our study did not consider other adverse life events, our cohort only included European-based players and data collection was partly done in the COVID-19 pandemic and these factors may have influenced our MHS prevalence findings. All screening tools relied on self-reporting of symptoms, and with MHS having a poor social stigma, under- or over-reporting may have occurred. Incidence measured over a longer period than 12 months may produce different results as follow-up responses from participants may be lost due to player movements between clubs.

## Conclusion

Based on this preliminary data, this study indicates that the likelihood of MHS in active professional male footballers is greater than in the global population and other sports. We found that at baseline and 12-month follow-up, the odds of an active professional male footballer developing MHS are greater after sustaining a severe injury and/or having surgery compared to those players who have not. This warrants the importance of providing mental health support to any player suffering from severe injury and/or surgery.

## Electronic supplementary material

Below is the link to the electronic supplementary material.

Supplementary Material 1

## Data Availability

At a reasonable request, data can be made available from the corresponding author. However, all collected data and analysis are presented in the text and tables.

## Declarations

### Conmpeting interests

None declared by any of the authors.
