# Supplementary material for: Don’t forget to mind the mind: a prospective cohort study over 12 months on mental health symptoms in active professional male footballers
Source: BMC Sports Sci Med Rehabil. 2024 Oct 14;16:214. doi: 10.1186/s13102-024-01005-1 (PMC11472507; doi:10.1186/s13102-024-01005-1)
Supplement: Supplementary file 1 — Supplementary Material 1 [file 13102_2024_1005_MOESM1_ESM.docx]

Baseline questionnaire

1. What is your gender?
2. How old are you?
3. What is your height?
4. What is your weight?
5. What level of education do you have?
   1. No school
   2. Nursery/elementary school
   3. High school
   4. University/vocational/other
6. Are you studying?
7. How many hours do you spend studying?
8. Are you working?
9. What work do you do?
10. How many hours do you spend working?
11. What field position do you play?
    1. Goalkeeper
    2. Defender
    3. Midfielder
    4. Forward
12. What level of football do you compete at?
    1. Highest national level
    2. Second highest national level
    3. Other
13. How many seasons have you played as a professional footballer?
14. Have you been admitted to a hospital?
15. What was the reason for your admission?
16. Have you been diagnosed with a mental health disorder?
17. If yes, what have you been diagnosed with?
18. Do you have family members diagnosed with a mental health disorder?
19. What mental health disorder have first degree relative/s been diagnosed with?
20. What mental health disorder have second degree relative/s been diagnosed with?
21. How many cigarettes have you smoke your entire life?
22. How many cigarettes have you smoked in the last 30 days?
23. How often do you use prescription pain medication?
    1. Do not use
    2. Yearly or less
    3. Monthly
    4. Weekly
    5. Daily
24. How often do you use over the counter pain medication?
    1. Do not use
    2. Yearly or less
    3. Monthly
    4. Weekly
    5. Daily
25. How often do you use prescription sleeping medication?
    1. Do not use
    2. Yearly or less
    3. Monthly
    4. Weekly
    5. Daily
26. How often do you use over the counter sleeping aids?
    1. Do not use
    2. Yearly or less
    3. Monthly
    4. Weekly
    5. Daily
27. How often do you use antidepressant medication?
    1. Do not use
    2. Yearly or less
    3. Monthly
    4. Weekly
    5. Daily

A severe injury is defined as an injury that resulted in >28 consecutive days of training or game time loss.

1. How many severe left hip injuries have you sustained?
2. How many severe right hip injuries have you sustained?
3. How many left hip surgeries have you undergone?
4. How many right hip surgeries have you undergone?
5. How many severe left knee injuries have you sustained?
6. How many severe right knee injuries have you sustained?
7. How many left knee surgeries have you undergone?
8. How many right knee surgeries have you undergone?
9. How many severe left ankle injuries have you sustained?
10. How many severe right ankle injuries have you sustained?
11. How many left ankle surgeries have you undergone?
12. How many left ankle surgeries have you undergone?

Follow up questionnaire

1. How many severe left hip injuries have you sustained?
2. How many severe right hip injuries have you sustained?
3. How many left hip surgeries have you undergone?
4. How many right hip surgeries have you undergone?
5. How many severe left knee injuries have you sustained?
6. How many severe right knee injuries have you sustained?
7. How many left knee surgeries have you undergone?
8. How many right knee surgeries have you undergone?
9. How many severe left ankle injuries have you sustained?
10. How many severe right ankle injuries have you sustained?
11. How many left ankle surgeries have you undergone?
12. How many left ankle surgeries have you undergone?
